# High Rates of Pneumonia in Children under Two Years of Age in a South East Asian Refugee Population

**DOI:** 10.1371/journal.pone.0054026

**Published:** 2013-01-08

**Authors:** Claudia Turner, Paul Turner, Verena Carrara, Kathy Burgoine, Saw Tha Ler Htoo, Wanitda Watthanaworawit, Nicholas P. Day, Nicholas J. White, David Goldblatt, François Nosten

**Affiliations:** 1 Shoklo Malaria Research Unit, Mae Sot, Thailand; 2 Mahidol-Oxford Tropical Medicine Research Unit, Bangkok, Thailand; 3 Centre for Tropical Medicine, University of Oxford, Oxford, United Kingdom; 4 Immunobiology Unit, Institute of Child Health, University College London, London, United Kingdom; Universidade Federal do Acre (Federal University of Acre), Brazil

## Abstract

**Background:**

There are an estimated 150 million episodes of childhood pneumonia per year, with 11–20 million hospital admissions and 1.575 million deaths. Refugee children are particularly vulnerable, with poorly defined pneumonia epidemiology.

**Methods:**

We followed a birth cohort of 955 refugee infants, born over a one-year period, until two years of age. Clinical and radiographic pneumonia were diagnosed according to WHO criteria. Detailed characteristics were collected to determine risk factors for clinical, radiological and multiple episodes of pneumonia. Investigations were taken during a pneumonia episode to help determine or to infer an aetiological diagnosis.

**Findings:**

The incidence of clinical pneumonia was 0.73 (95% CI 0.70–0.75) episodes per child year (/CY) and of radiological primary endpoint pneumonia (PEP) was 0.22/CY (95% CI 0.20–0.24). The incidence of pneumonia without severe signs was 0.50/CY (95% CI 0.48–0.53), severe pneumonia 0.15/CY (95% CI 0.13–0.17) and very severe pneumonia 0.06/CY (0.05–0.07). Virus was detected, from a nasopharyngeal aspirate, in 61.3% of episodes. A reduced volume of living space per person (IRR 0.99, 95% CI 0.99–1.0, p = 0.003) and young maternal age (IRR 1.59, 95% CI 1.12–2.27, p = 0.01) were risk factors for developing pneumonia. The risk of a child having >1 episode of pneumonia was increased by having a shorter distance to the next house (IRR 0.86, 95% CI 0.74–1.00, p = 0.04). Infants were at risk of having an episode of PEP if there was a shorter distance from stove to bed (IRR 0.89, 95% CI 0.80–0.99, p = 0.03). Raised CRP and neutrophil values were associated with PEP.

**Conclusions:**

There was a high incidence of pneumonia in young children in this SE Asian refugee population. Viral infections were important, however CXR and non-specific marker findings suggested that bacteria may be involved in up to a third of cases.

## Introduction

The UNHCR estimates that there are 15 million refugees in the world, a third of whom live in crowded camps and approximately half are children [1]. Refugees are at high risk from increased morbidity and mortality from acute respiratory infections. However in these populations there is a paucity of data on the epidemiology of pneumonia [2].

Pneumonia contributes significantly to global childhood morbidity and mortality. It is estimated to cause 1·575 million deaths per year in children less than five years old, more deaths than any other disease [3]. The majority of these deaths (70%) occur in Africa and South East Asia [4]. These pneumonia deaths represent just the tip of a larger disease iceberg: there are an estimated 150 million episodes of childhood pneumonia per year with 11–20 million of these needing hospital admission [5]. The global incidence of clinical pneumonia in the under-five population is reported to be 0.28 (IQR: 0.21–0.71) episodes per child year with 95% of cases occurring in the developing world [6]. The incidence of pneumonia in South East Asia is estimated to be 0.36 (IQR: 0.32–0.40) episodes per child year, however data is limited for many areas of the region [7].

The fourth millennium development goal aims to reduce the under-five childhood mortality by two thirds by 2015 [8]. In order to achieve this, the number of deaths from pneumonia must be reduced and for this robust regionally specific epidemiology data are required. The diagnosis of childhood pneumonia is difficult and often depends upon clinical opinion. The WHO/UNICEF Integrated Management of Childhood Illness (IMCI) guideline includes a standardised definition of pneumonia based on clinical signs: cough or difficulty breathing and a rapid respiratory rate, with additional signs determining severity [9]. Using this standard definition to calculate pneumonia incidence, data can be compared globally. Confirmation of the etiological agent is yet another challenge and radiological changes are often used to infer bacterial aetiology. The standard definition of radiological pneumonia, developed by the WHO permits epidemiological data to be compared across studies. This definition specifies four diagnoses which can be made – primary endpoint pneumonia (PEP), other infiltrate (OI), normal, or uninterpretable [10].

Viral infections have been identified as the etiological agents in 20–40% of children hospitalized with pneumonia [11], [12]. Indeed it has been stated that viruses cause more episodes of acute lower tract infections than bacteria [13]. An estimated 100 million episodes of viral community acquired pneumonia occur in children each year, potentially up to two thirds of the total pneumonia disease burden [14].

The aims of this study were to determine the epidemiology of pneumonia in children less than two years of age in a South East Asian refugee population, explore the relative contributions of bacterial and viral pathogens, and to assess the risk factors for developing pneumonia. The under two year old age group was chosen as this is where the burden of pneumonia was likely to be highest [15], [16].

## Methods

### Setting

Maela camp for displaced persons is located 5km east of the Thailand-Myanmar (Burma) border, ∼500 km Northwest of Bangkok, predominantly inhabited by refugees of Karen ethnicity. It is densely populated with an estimated 10,000 houses in 4 km^2^. Approximately 45,000 people live in the camp, and 14% are under five years old [17].

The majority of houses in Maela are constructed from natural materials such as bamboo and leaves. Indoor charcoal-burning stoves are used for cooking and registered refugees receive standard rations provided by the Thailand Burma Border Consortium (www.tbbc.org).

The climate is tropical with temperatures ranging from 15°C–45°C and the monsoon occurring from May to October. Seasons are defined as being hot (March – May), wet (June – October) and cool (November – February).

Since 1986 the Shoklo Malaria Research Unit (SMRU) has provided medical and obstetric care for this population. There are approximately 1,500 deliveries each year in the camp and 90% of pregnant women attend SMRU's antenatal clinics. Although Premiere Urgence-Aide Médicale Internationale (PU-AMI) provides general medical care for the camp, SMRU provided the medical care for children in the study, using guidelines based on WHO recommendations [9], [18]. Local healthcare workers were trained in the use of these guidelines prior to the study. Standard Expanded Programme on Immunisations vaccinations are provided to all children living in the camp, with excellent coverage (93% of all children who reached two years in the reported cohort had all planned vaccines). Hib and pneumococcal conjugate vaccines are not available.

### Study design

A cohort of infants was followed from birth until 24 months of age. Pregnant women, who were receiving antenatal care at SMRU, were asked to participate in the study at 28–30 weeks of gestation. Enrolment commenced in September 2007 and was completed in September 2008. At the time of delivery a questionnaire was completed by mothers to determine household composition, ethnic group, malaria during pregnancy, fuel used in the house and incidence of smoking. After delivery the families were asked to return to the clinic for follow up visits once a month for the duration of the study period. At these visits assessment of health status and growth were performed. Parents were also asked to return when their infant was unwell, and specifically if their child had cough or difficulty breathing. Weekly visits were made to the PU-AMI hospital to capture any other illness episodes.

### Pneumonia episodes

During an illness visit, pneumonia was diagnosed using WHO criteria and its severity graded [18]. Children with cough or difficulty breathing and a fast respiratory rate, as determined by age specific cut offs (<2 months ≥60 breaths per minute; 2 months –1 year ≥50 breaths per minute and >1 year ≥40 breaths per minute), but no severe signs were diagnosed as having pneumonia. Severe pneumonia was diagnosed in children with cough or difficulty breathing and chest indrawing. Children fulfilling the severe pneumonia criteria but who also had cyanosis or inability to suck were diagnosed with very severe pneumonia.

Pulse oximetry (Hand –held pulse oximeter 512, Respironics), a complete blood count (CBC) (PocH-one 100i, Sysmex), C-reactive protein (CRP) (NycoCard, Axis-Shield), nasopharyngeal aspirate (NPA), and a chest radiograph (CXR) were done on all children with diagnosed pneumonia. NPAs were tested for influenza viruses, respiratory syncytial virus (RSV), human metapneumovirus (hMPV) and adenovirus by rRT-PCR [19].

CXRs were interpreted by two clinicians using the WHO criteria [10]. Results were compared and any conflicting CXRs were examined by a third clinician and the majority diagnosis was used. Where there was no agreement the CXR was deemed uninterpretable.

Pneumonia treatment followed the recommendations in the WHO pocket book of hospital care for children [18].

### Additional data

A household survey of all participants was performed: data were collected on household composition and structure. A questionnaire was administered to assess the degree of indoor air pollution and measurements were taken to calculate the floor area and volume of each house. The volume of each house was divided by the number of occupants to determine the volume of living space per person.

### Data management and statistical methods

All data were double entered and checked systematically for errors prior to analysis using Stata/IC 12.1 (StataCorp). Comparisons of continuous variables between groups were made using the Wilcoxon rank sum test. Proportions were compared using Chi-squared or Fisher's exact test as appropriate. Incidence rates were analysed using Poisson regression and groups compared by incidence rate ratios (IRR). Odds ratios were calculated using logistic regression. For univariate analysis of potential risk factors, two by two tables were constructed and association was tested by the chi-squared test. Factors with a significant p-value (<0.05) were included in a multivariate logistic regression model.

### Ethical approval

Ethical approval was granted by the Ethics Committee of The Faculty of Tropical Medicine, Mahidol University, Thailand (MUTM 2009-306) and the Oxford Tropical Research Ethics Committee, Oxford University, UK (031-06). All women gave written informed consent to participate in the study.

## Results

Nine hundred and ninety nine women were enrolled into the study, including 991 singleton and eight twin pregnancies. Forty two mothers were lost to follow-up during their pregnancy or in the first month after the estimated date of delivery. There were 965 births into the study, 955 of which were live births. Twenty five infants died in the first month giving a neonatal mortality rate (NMR) of 26 per 1,000 live births. During the study there was a planned relocation of refugees to the USA which increased losses from the cohort: 154 infants left the study in the first year of life and 136 left in the second year. The characteristics of the cohort are summarized in Table 1.

**Table 1:** Summary of the cohort characteristics.

Characteristic	Number
**Sex**	
Male (%)	484 (51)
Female (%)	471 (49)
**Birth weight (kg)**	
Mean (SD)	2.92 (0.47)
<2.50 kg (%)	139 (14.7)
**Gestation (weeks)**	
Mean (SD)	39 (2)
Premature (<37 completed weeks) (%)	79 (8.3)
**Delivery**	
Home (%)	180 (18.9)
Duration of pre-partum rupture of membranes (hours), median (range)	1 (0–240)
Prolonged rupture of membranes (> 18hours) (%)	30 (4.3%)
**Season of birth**	
Wet (%)	391 (40.9)
Hot (%)	205 (21.5)
Cool (%)	359 (37.6)
**Ethnic group**	
Sgaw Karen (%)	653 (69.2)
Paw Karen (%)	125 (13.2)
Muslim (%)	121 (12.8)
Other (%)	45 (4.8)
**Mothers age**	
Age in years, mean (SD)	26 (6.7)
<20 years (%)	162 (17.1)
<18 years (%)	63 (6.7)
**Gravidity and parity**	
Median gravida (range)	3 (1–17)
Median parity (range)	2 (1–11)
**Household**	
Number of people living in house (including study infant), median (range)	5 (2–15)
<5 years (not including study infant) (range)	1 (0–4)
5–15 years (range)	1 (0–9)
15–60 years (range)	3 (1–12)
>60 years (range)	0 (0–2)
Households with members >60 years (%)	101 (13.4)
Number of rooms in the house, median (range)	2 (1–7)
Number of people sleeping in the same bed as the study infant (excluding the study infant), median (range)	3 (1–8)
Number of mothers who smoked (%)	256 (27.1)
Number of smokers in the household, median (range)	1 (0–6)
Animals kept by family (%)	340 (45)
Distance to next house in m, median (range)	1.97 (0–270)
**Cooking stove**	
Poor ventilation reported in the house (%)	241 (32)
Distance from stove to infants bed in m, median (range)	4.4 (0.6–11.6
**House size**	
Floor area in m^2^, median (range)	34.8(2.2–136.2)
House volume in m^3^, median (range)	101.0 (6.0–626.5)
House volume per person in m^3^, median (range)	19.1 (0.9–313.3)

### Pneumonia episodes

There were a total of 1,085 clinical episodes of pneumonia and 1,494.9 child years at risk, giving a pneumonia incidence rate of 0.73 (95% CI 0.70–0.75) episodes per child year. Approximately half the children (488/955; 51·1%) had an episode of pneumonia. Of these the median number of pneumonia episodes per child was three [range 1–12]. Further examining WHO pneumonia categories, the incidence of pneumonia without severe signs was 0.50 (95% CI 0.48–0.53) episodes per child year, severe pneumonia 0.15 (95% CI 0.13–0.17) episodes per child year and very severe pneumonia 0.06 (0.05–0.07) episodes per child year.

A total of 1,020 CXRs were taken, 989 (97.0%) of which were interpretable. The most common CXR finding was OI (552/1,020 54.1%) an incidence of 0.37 per child year at risk. PEP was found on 331/1,020 (32.4%) of the CXRs, giving an incidence of PEP of 0.22 per child year at risk. PEP was found more commonly in children >12 months of age (p<0.001). Children with more severe clinical disease were more likely to have an abnormal CXR (p = 0.01) (Table 2). Only one child died from pneumonia giving an incidence of 1.0 per 1,000 children (95% CI 0.03–5.82).

**Table 2:** Breakdown of radiological diagnosis by clinical disease and WHO age categories.

Age group	Pneumonia (% of CXR diagnosis)	Severe pneumonia (% of CXR diagnosis)	Very severe pneumonia (% of CXR diagnosis)	Total
<2 months	6	15	12	
PEP	0	3 (75.0)	1 (25)	4
OI	2 (10.5)	7 (36.8)	10 (52.6)	19
Normal	4 (40.0)	5 (50.0)	1 (10.0)	10
2–11 months	373	105	36	
PEP	103 (66.5)	36 (23.3)	16 (10.3)	155
OI	196 (71.5)	58 (21.2)	20 (7.3)	274
Normal	74 (87.1)	11 (12.9)	0	85
≥12 months	331	87	24	
PEP	121 (70.4)	40 (23.3)	11 (6.4)	172
OI	201 (77.6)	45 (17.4)	13 (5.0)	259
Normal	9 (81.8)	2 (18.2)	0	11
All	710 (71.8)	207(20.1)	72 (7.3)	989

### Viruses

**Figure 1:**
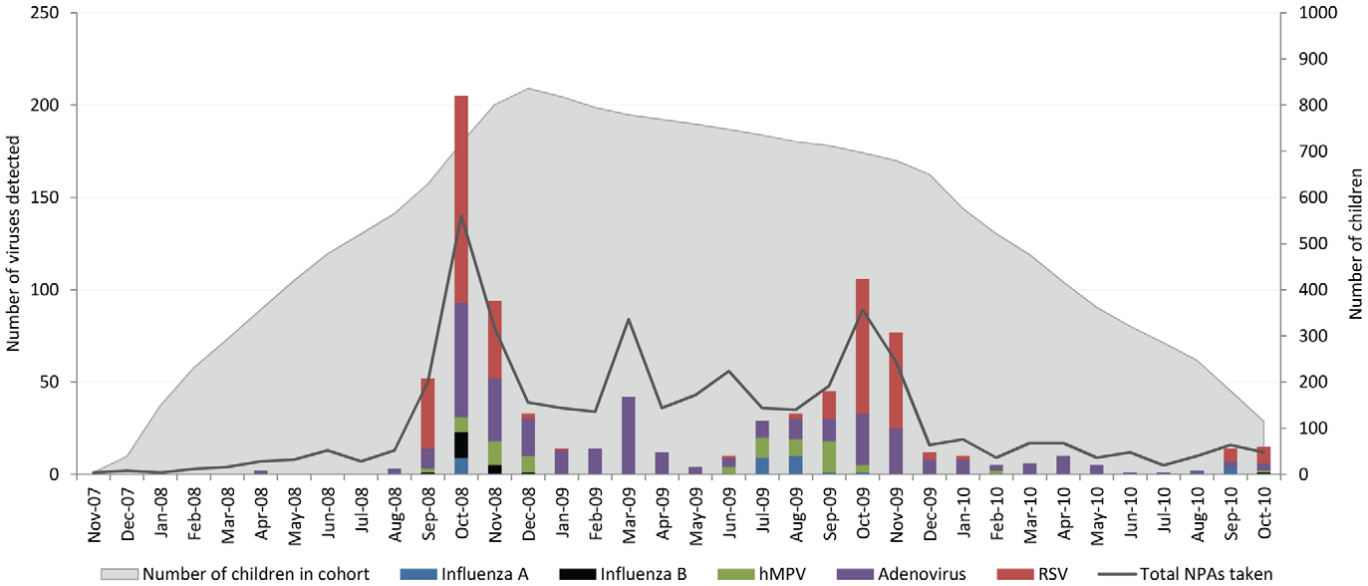
Virus detection by month of year and total number of NPAs taken.

Viruses were detected in NPA specimens in 654/1,067 (61.3%) pneumonia episodes. Detection of multiple viruses was not uncommon: 174/1,067 (16.3%) were positive for two viruses and 14/1,067 (1.3%) were positive for three viruses. The most commonly detected virus was RSV, in 362/1,067 (33.9%) of cases. RSV detection was significantly associated with a radiological diagnosis of OI (p = 0.001) and was not associated with PEP (p = 0.2). There was no association between any of the viruses and a radiologic diagnosis of PEP. However, RSV was identified in 31.0% of PEP cases. These cases were more likely to have a high neutrophil count and a high CRP, compared to other pneumonia cases where RSV was detected (OR 2.3, 95% CI 1.5–3.5, p<0.001), implying that these cases represented a bacterial superinfection of RSV associated pneumonia. Pneumonia and identification of virus was highly seasonal (Figure 1).

### Non-specific markers of infection

A blood sample for CRP was taken in 95.4% of all pneumonia episodes; the median value was 14.4 mg/L (IQR 8–200 mg/L). 184/1,035 (17.8%) pneumonia episodes had a CRP ≥40 mg/L. During a pneumonia episode a CRP ≥40 mg/L was associated with PEP on CXR (p<0.001) but not with OI (p = 0.004).

A CBC was taken in 96.5% of pneumonia cases. Controlling for RSV detection there was no association between having a high WBC (defined as >15×10^9^/L) and having PEP on the CXR (p = 0.09). There was however an association between a high WBC and having very severe pneumonia, when controlling for RSV detection (OR 1.7, 95% CI 1.0–2.8, p = 0.04). Lymphopenia (defined as <15% of the total WBC) was only seen in 10/1,045 (0.1%) of pneumonia episodes. Pneumonia episodes where PEP was present on CXR were more likely to have a high neutrophil count (defined as >7.5×10^9^/L) (p = 0.02).

Controlling for age, children with high CRP and neutrophil count had a significantly higher risk of PEP when compared to other CXR findings (p<0.001). Controlling for RSV detection, severe clinical disease was not associated with a high CRP and neutrophil count (p = 0.09) (Table 3).

**Table 3:** Summary of multivariate logistic regression analyses of risk factors for clinical and radiological pneumonia.

	Neutrophils >7.5×10^9^/L & CRP ≥40mg/L
**Very Severe Pneumonia**	
Odds Ratio (95% CI)	1.66 (0.85–3.24)
p-value	0.09
**Primary Endpoint Pneumonia**	
Odds Ratio (95% CI)	2.37 (1.56–3.59)
p-value	<0.001
**Other Infiltrate**	
Odds Ratio (95% CI)	0.54 (0.36–0.81)
p-value	0.003

Episodes of pneumonia categorised clinically as very severe pneumonia, radiologically as primary endpoint pneumonia or other infiltrate were compared to all other pneumonia episodes.

The combination of a high CRP and neutrophil count had a specificity of 89.7% (95% CI 92.1–94.1), but a sensitivity of only 13.3% (95% CI 17.4–22.0), to predict PEP on CXR.

### Risk factors

The characteristics in Table 1 were analysed to determine their relationship to risk of clinical pneumonia, having multiple episodes of pneumonia or having PEP on CXR. Having a mother <18 years old (p = 0.01), a smaller living space per person (p = 0.03) and a smaller number of rooms in the house (p = 0.004) were found to significantly increase the risk of developing pneumonia in the first two years of life. The risk of having multiple episodes of pneumonia was increased by having a shorter distance to next house (p = 0.04). Infants were at increased risk of having an episode of PEP if there was a shorter distance from stove to infant bed (p = 0.03) (Table 4).

**Table 4:** Significant results from a multivariate Poisson regression model assessing potential risk factors for pneumonia.

Risk factor	IRR	95% CI	p-value
**First episode of pneumonia** a			
Maternal age <18 years	1.59	1.12–2.27	0.01
House volume per person (m3)	0.99	0.99–1.00	0.03
Number of rooms	0.85	0.77–0.95	0.004
**Multiple episodes of pneumonia** b			
Distance to next house	0.86	0.74–1.00	0.04
**Primary endpoint pneumonia** c			
Distance stove to bed	0.89	0.80–0.99	0.03

In addition to the factors in the table, the multivariate model included all factors from Table 1 which were significant in univariate analysis. The following were non-significant:

a distance from the household stove to the infants sleeping area.

b season of birth.

c birth weight and the number of people sleeping in the same bed as the infant.

## Discussion

The observed incidence of pneumonia in this cohort (0.74 episodes per child year) is high. A strength of our study is that we robustly followed WHO definitions for clinical and radiological pneumonia. Using standardized definitions allows accurate comparisons to be made between studies. The Pneumonia Etiology Research for Child Health Project (PERCH) is a standardized multi-centre study of the aetiology of pneumonia and is an excellent example of a study where direct comparisons could be made to our study [20].

The reported NMR was 26 per 1000 live births, a figure which lies between that reported from Thailand and Burma (NMR 8 versus 32 per 1000 live births).

We followed a cohort of infants born and living in a refugee camp where the living conditions are crowded and indoor air pollution is common. Because of their nature there are many challenges in studying refugee and crisis affected populations. Between 2010 and 2011, UNHCR reported a global increase in refugees of 700,000, a trend which is, unfortunately, likely to continue to increase. A systematic review of the burden of acute respiratory infections in crisis-affected populations reports on only 36 studies and only one of these reported community incidence. The authors call for further epidemiological studies to improve medical care for these populations. Our study is therefore an important addition to the literature [1], [2].

The use of CXR changes to predict the aetiology of pneumonia is controversial. Some studies have concluded that radiographic evidence of consolidation was a reliable indicator of bacterial pneumonia [21]. However a study from The Gambia found that only 49.1% children with radiographic evidence of consolidation had a confirmed bacterial infection [22]. The WHO aimed to standardize the interpretation of CXRs with the publication of its definitions for radiological pneumonia. Significant alveolar consolidation was chosen as the primary end point as it is was considered to be the most specific radiological predictor of a bacterial aetiology [23]. In this study we found that approximately one third of children with pneumonia had a diagnosis of PEP. This result is comparable to other pneumonia studies which used the WHO CXR definitions [24].

Non-specific markers of infection have been investigated in numerous studies to determine their utility in diagnosing bacterial pneumonia. Following the pneumococcal conjugate vaccine trial in South Africa, the usefulness of CRP as a measure of vaccine efficacy was compared to CXR diagnosis. It was concluded that CRP levels ≥40 mg/L provided a better measure than CXR in assessing vaccine efficacy [25].

Total WBC has also been evaluated for use in determining bacterial aetiology. However it has not been found to be sensitive or specific enough for use in the confirmation of bacterial pneumonia [26]. In this study we studied the absolute neutrophil count, as neutrophils respond rapidly to bacterial infection. The reported upper limit of normal in children less than two years of age is 7.5×10^9^/L and this was used in our study [27]. We found that the combination of a high CRP (≥40 mg/L) and a high neutrophil count was significantly associated with PEP and was not associated with OI on radiograph or the detection of RSV, strengthening the hypothesis of a bacterial aetiology in our cases of PEP. The combination of a high CRP and neutrophil count had good specificity in determining PEP on CXR but a low sensitivity, making it an unsuitable test to diagnose pneumonia. It may, however, be useful in determining which of the children who fulfil the WHO criteria for pneumonia do not need to receive antibiotics.

Viruses are reported to be a significant cause of childhood pneumonia [28]. In this study we found that a potentially causative virus was detected by PCR in nearly two thirds of pneumonia cases. RSV was the most commonly detected virus and was highly seasonal. Interestingly, RSV was detected in one third of all cases with PEP on CXR, which potentially implies bacterial superinfection in these cases, as was demonstrated in the South African pneumococcal conjugate vaccine trial [29].

Although the definitive cause of pneumonia in the cohort studied cannot be determined, it is possible, with the use of CXR findings, nonspecific markers and nasopharyngeal virus detection to conclude that the highest burden of disease was due to viruses, but that bacteria also contributed significantly both as primary etiological agents and through possible secondary infection following a viral infection.

Crowding and indoor pollution are known to be risk factors for pneumonia [30]. However ours was the first study to quantify this by assessing the volume of living space per person as a risk factor for pneumonia. We found that having a smaller living space was significantly associated with an increased risk of developing pneumonia in the first two years of life.

The protective effect of breast feeding and the impact of HIV infection could not be examined in the study infants as >99% of infants in the cohort were breast fed and only one (0.1%) mother had HIV infection.

## Conclusions

We found a high incidence of pneumonia in children less than two years of age living in a refugee population. Crowding and indoor pollution were likely contributors. Viruses played an important role in the aetiology of pneumonia; however CXR findings and non-specific markers suggested that bacteria were involved in up to a third of pneumonia cases.
